# Supplementary material for: Chemical Identification of Secondary Metabolites from Rhizospheric Actinomycetes Using LC-MS Analysis: In Silico Antifungal Evaluation and Growth-Promoting Effects
Source: Plants (Basel). 2023 May 2;12(9):1869. doi: 10.3390/plants12091869 (PMC10181443; doi:10.3390/plants12091869)
Supplement: Supplementary file 1 [file plants-12-01869-s001.zip › plants-2359935-supplementary.pdf]

# Supplementary Material

# Chemical Identification of Secondary Metabolites from Rhizospheric Actinomycetes Using LC-MS Analysis: In Silico Antifungal Evaluation and Growth-Promoting Effects

**Hazem S. Elshafie [1], Laura De Martino [2], Carmen Formisano [3], Lucia Caputo [2], Vincenzo De Feo [2] and Ippolito Camele [1,*]**

[1] School of Agricultural, Forestry, Food and Environmental Sciences, University of Basilicata, 85100 Potenza, Italy; hazem.elshafie@unibas.it
[2] Department of Pharmacy, University of Salerno, Via Giovanni Paolo II 132, 84084 Fisciano, Italy; ldemartino@unisa.it (L.D.M.); lcaputo@unisa.it (L.C.); defeo@unisa.it (V.D.F.)
[3] Department of Pharmacy, School of Medicine and Surgery, University of Naples Federico II, Via Montesano 49, 80131 Naples, Italy; carmen.formisano2@unina.it (C.F.)
* Correspondence: ippolito.camele@unibas.it; Tel.: +39-0971-205544; Fax: +39-0971-205503 (I.C.)

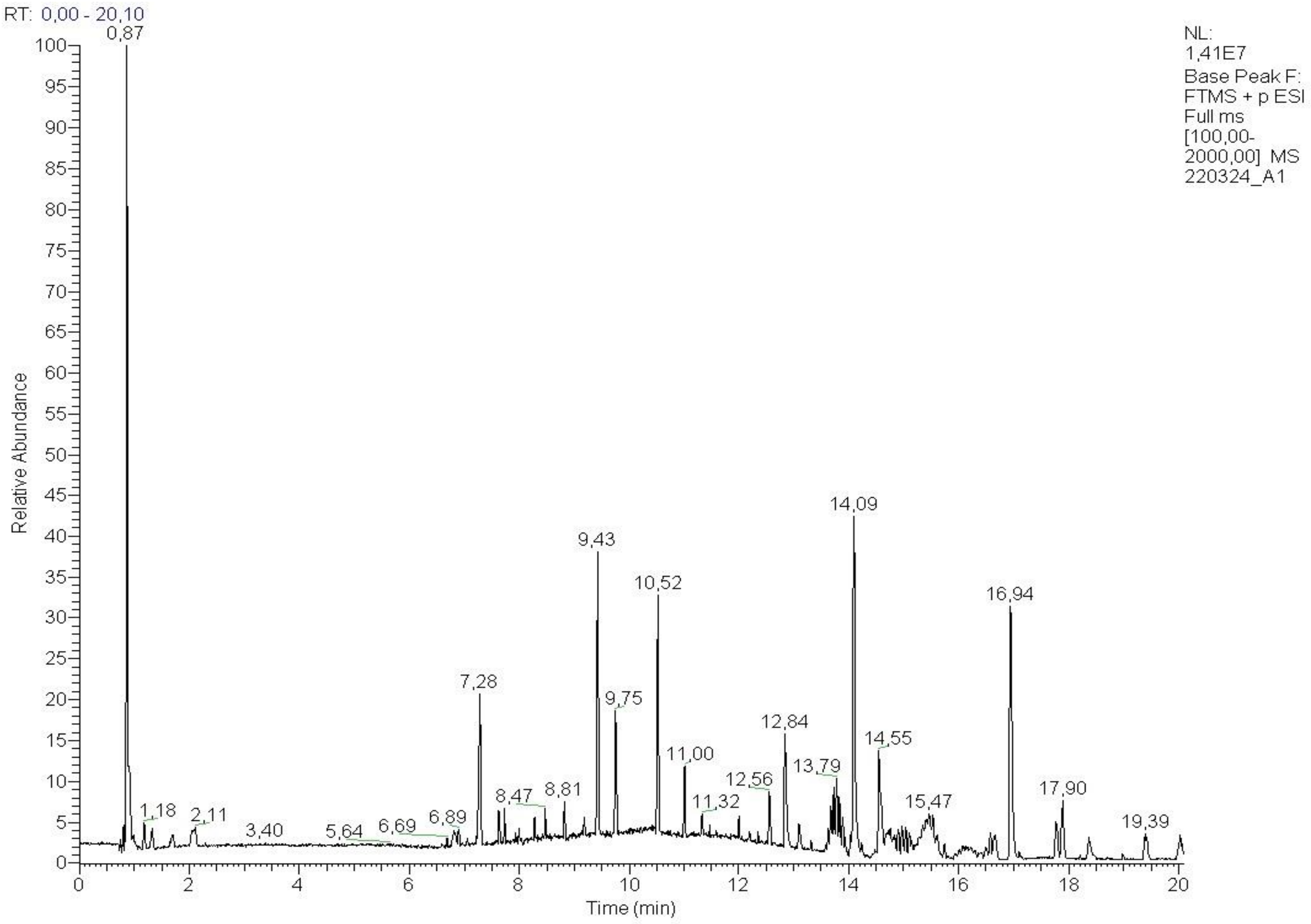


**Figure S1.** LC-MS chromatogram of the metabolites extracted from *Streptomyces* spp. (Act1).

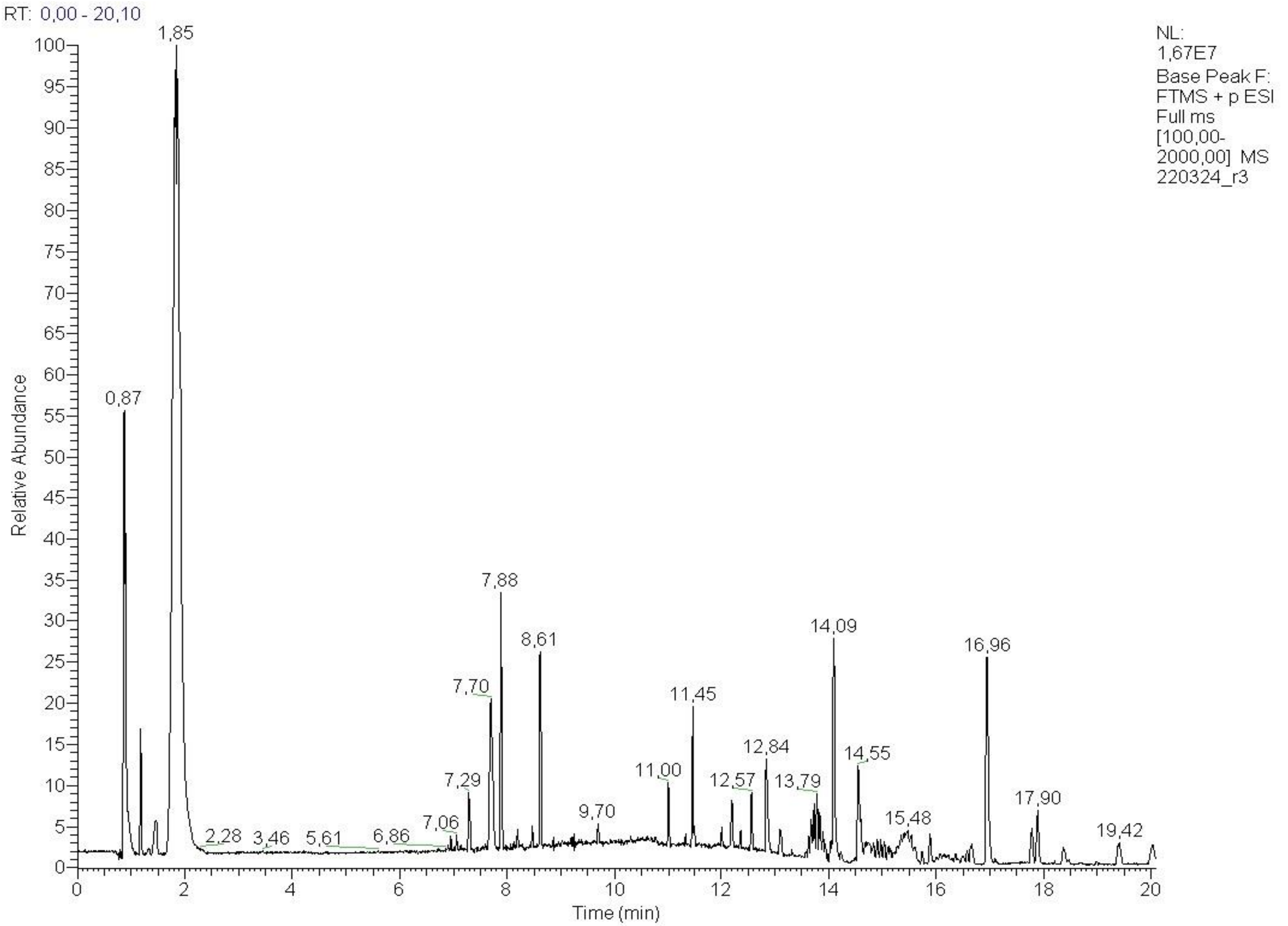


**Figure S2.** LC-MS chromatogram of the metabolites extracted from *S. atratus* (Act2).

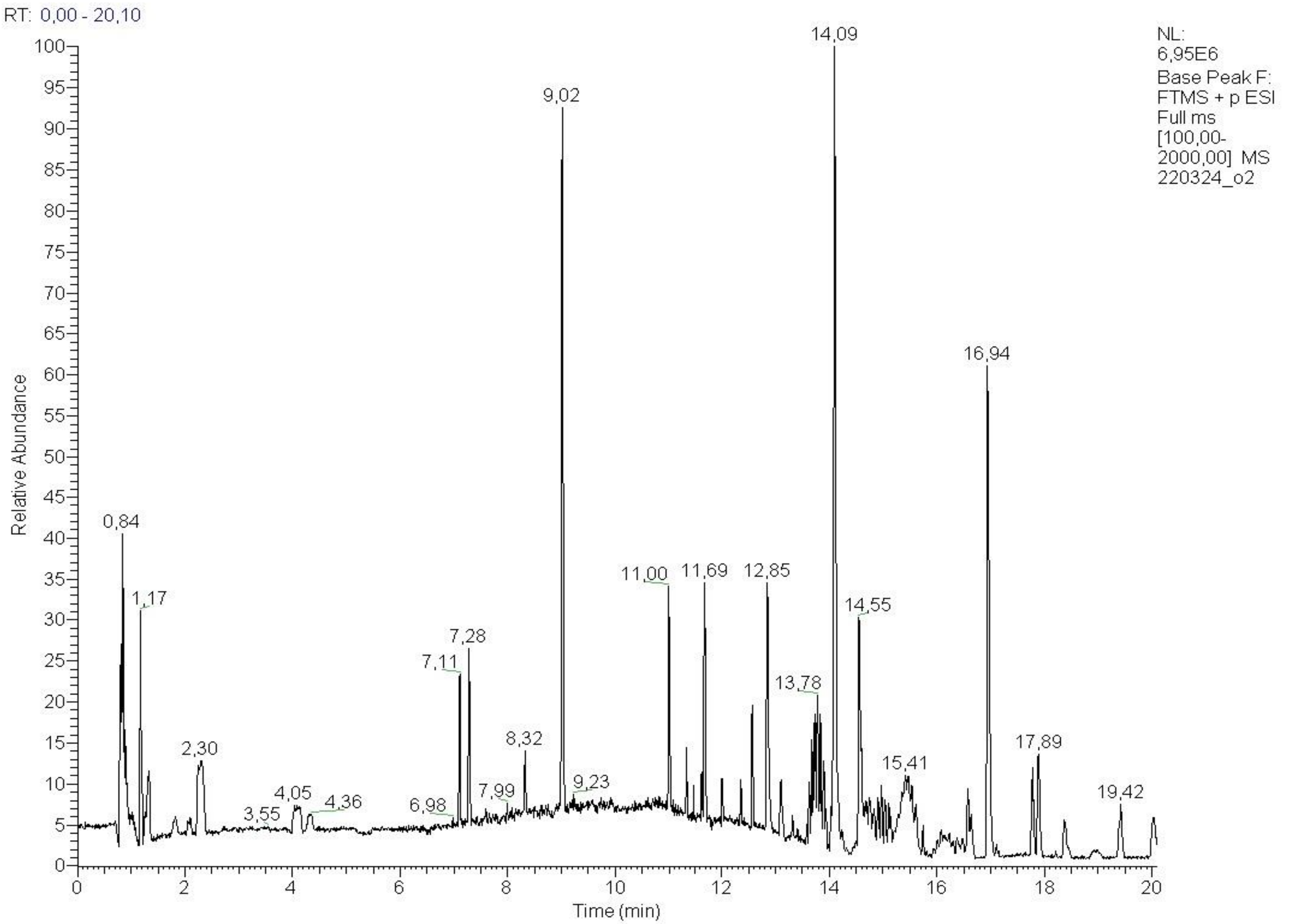


**Figure S3.** LC-MS chromatogram of the metabolites extracted from *A. humicola* (Act3).

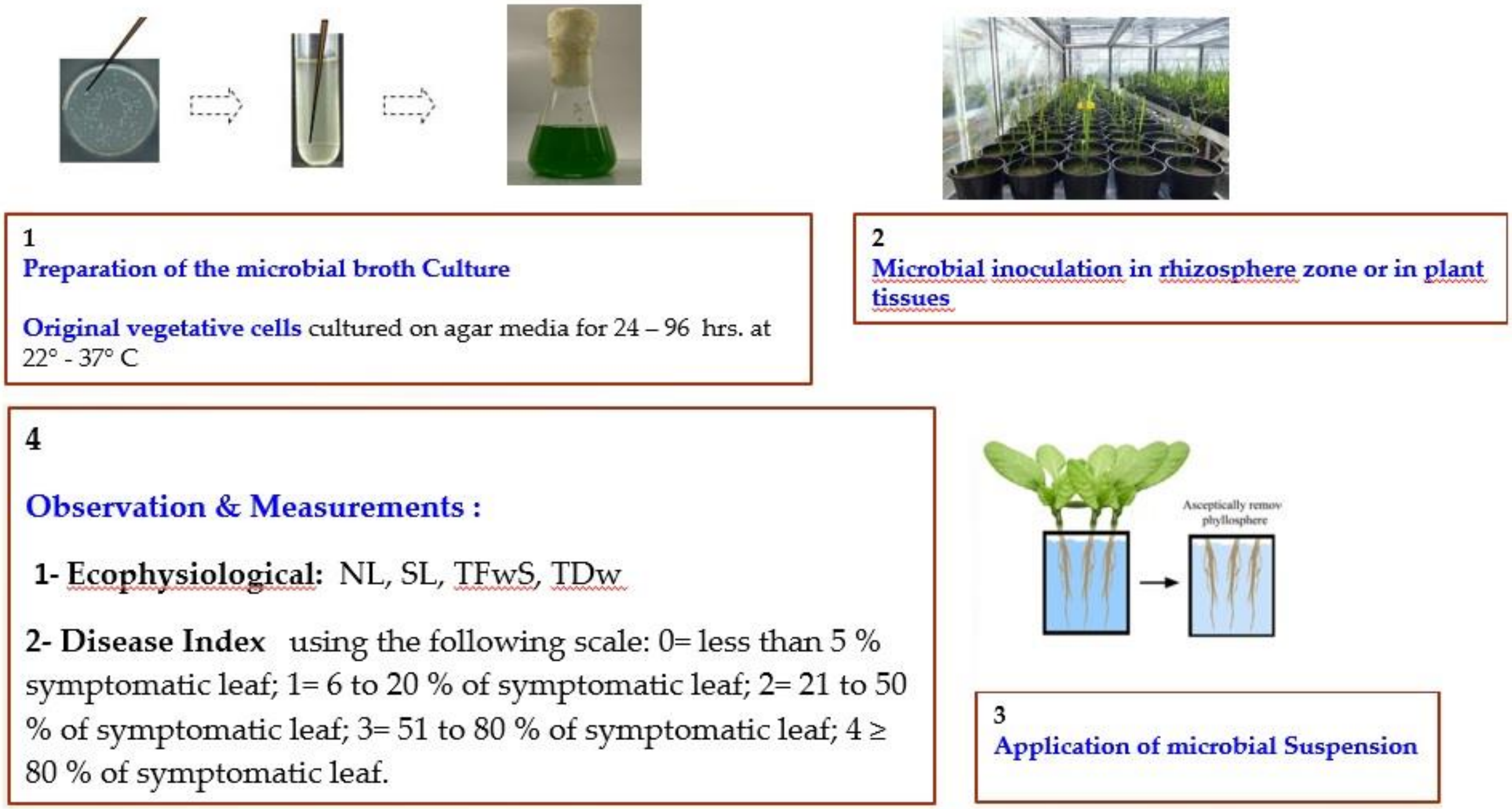


**Figure S4.** Methodological procedures of in silico experiment in greenhouse.
